# Association between Multidrug-Resistant Tuberculosis and Risk Factors in China: Applying Partial Least Squares Path Modeling

**DOI:** 10.1371/journal.pone.0128298

**Published:** 2015-05-28

**Authors:** Yun-Xia Liu, Chun-Kun Pang, Yanxun Liu, Xiu-Bin Sun, Xin-Xu Li, Shi-Wen Jiang, Fuzhong Xue

**Affiliations:** 1 Department of Epidemiology and Biostatistics, School of Public Health, Shandong University, Jinan, China; 2 Institute Office, Shandong Academy of Medical Science, Jinan, China; 3 Institute for Tuberculosis Control and Prevention, Chinese Center for Disease Control and Prevention, Beijing, China; Texas A&M University, UNITED STATES

## Abstract

**Background:**

Multidrug-resistant tuberculosis (MDR-TB) resulting from various factors has raised serious public health concerns worldwide. Identifying the ecological risk factors associated with MDR-TB is critical to its prevention and control. This study aimed to explore the association between the development of MDR-TB and the risk factors at the group-level (ecological risk factors) in China.

**Methods:**

Data on MDR-TB in 120 counties were obtained from the National Tuberculosis Information Management System, and data on risk-factor variables were extracted from the Health Statistical Yearbook, provincial databases, and the meteorological bureau of each province (municipality). Partial Least Square Path Modeling was used to detect the associations.

**Results:**

The median proportion of MDR-TB in new TB cases was 3.96% (range, 0–39.39%). Six latent factors were extracted from the ecological risk factors, which explained 27.60% of the total variance overall in the prevalence of MDR-TB. Based on the results of PLS-PM, TB prevention, health resources, health services, TB treatment, TB detection, geography and climate factors were all associated with the risk of MDR-TB, but socioeconomic factors were not significant.

**Conclusions:**

The development of MDR-TB was influenced by TB prevention, health resources, health services, TB treatment, TB detection, geography and climate factors. Such information may help us to establish appropriate public health intervention strategies to prevent and control MDR-TB and yield benefits to the entire public health system in China.

## Introduction

Tuberculosis (TB) is still the world's leading infectious cause of adult deaths and a major public health burden in developing countries [1,2]. In 2013, there were an estimated 9.0 million (range, 8.6–9.4 million) new TB cases and 1.5 million TB deaths globally [2]. According to the 2014 World Health Organization (WHO) global TB report, China ranks as second among the world’s 22 high-burden countries with a TB incidence of approximately 0.9–1.1 million, accounting for 11% of the total number of cases in 2013 [2].

The increasing prevalence of drug-resistant TB (DR-TB), especially multidrug-resistant TB (MDR-TB), is a serious threat to global TB control and has become a major public health concern in several countries [1,3]. Beginning in 1994, the WHO and the International Union Against Tuberculosis and Lung Disease (WHO/IUATLD) initiated the Global Project on Anti-TB Drug Resistance Surveillance [4]. Currently, 144 countries have been covered [5]. Since 1996, an increasing number of provinces (municipalities) in China have begun to conduct drug resistance surveillance based strictly on the WHO/IUATLD Guidelines [4,6]. China is one of 27 countries in the world with the highest burden of MDR-TB, there were estimated to be 5.7% (95% CI 4.5–7.0%) of new TB cases and 26% (95% CI 22–30%) of previously treated cases with MDR-TB according to WHO [2,4].

Identifying the risk factors contributing to the development of essential and may help in developing appropriate MDR-TB control strategies [7]. Previous studies have identified several individual-level risk factors for MDR-TB, including but not limited to age [7–9,10,11], sex [8], genetic susceptibility [12,13], occupation [7,14], previous treatment [7–11,15–22,8,9,10,], smoking [7,11], alcohol abuse [11], human immunodeficiency virus (HIV) infection [11], and socioeconomic status [13,15,23–25]. However, as an infectious disease, MDR-TB could be due not only to individual-level factors but also to group-level factors, such as TB case notification rates, health service, health expenditure, the directly observed therapy short course and climatic factors [26–28]. Nevertheless, to the best of our knowledge, few specialized studies in China exploring group-level MDR-TB risk factors have been published. Therefore, we conducted the current ecological study to explore the relationships between group-level risk factors and MDR-TB in China. This research can provide clues to the etiology of the disease and knowledge that can be utilized for potential control and prevention of the disease [29].

## Materials and Methods

### Study area

**Fig 1:**
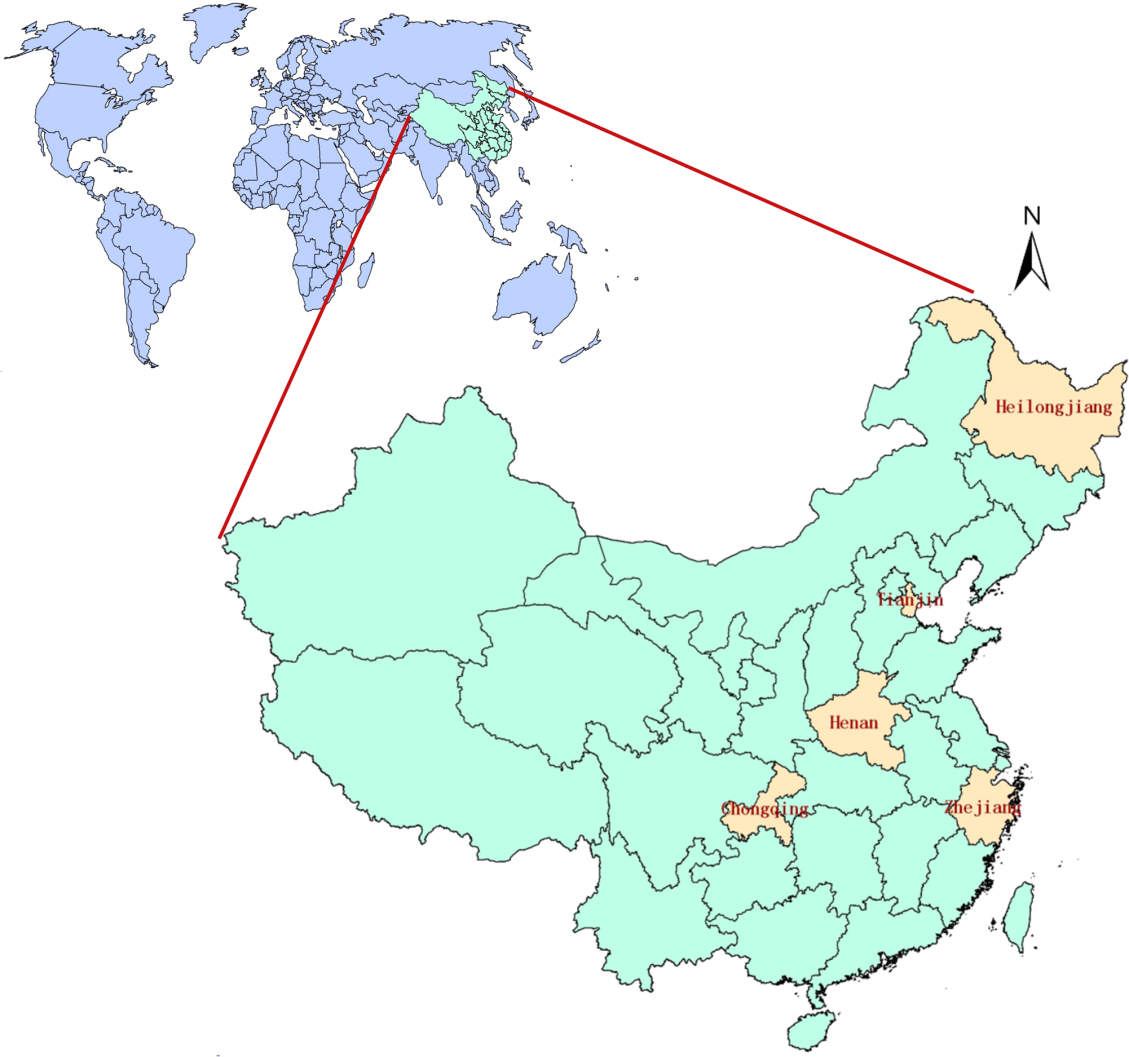
Location of the study area, showing Heilongjiang, Tianjin, Henan, Chongqing and Zhejiang provinces in China.

Based on a baseline survey of drug-resistant TB, the epidemic of TB, the prevention and treatment of TB, socioeconomic conditions, geographical locations, three provinces (Henan, Zhejiang and Heilongjiang) and two municipalities (Chongqing and Tianjin) were selected as regions of study. These provinces and municipalities lie in the east-central, eastern, northern, southwestern and north-central regions of China (Fig 1).

### Data sources

Data on first-line anti-TB drug resistance among new cases from the 120 counties surveyed were obtained from the National TB Information Management System [30] created by the Institute for Tuberculosis Control and Prevention of the Chinese Center for Disease Control and Prevention, which authorized us to use the data for this collaborative study. In addition, a database on ecological risk factors potentially related to MDR-TB was compiled, consisting of socioeconomic variables as well as other variables that pertained to health resource allocation, health services, TB prevention and treatment, climate and geography. The information for the study was obtained from the Health Statistical Yearbook, the provincial databases, and the meteorological bureau of each province (or municipality). In this study, we used an ecological study design to explore the association between MDR-TB and related risk factors, with the statistical unit being the drug resistance surveillance county. The data are at county level and include no personal information.

### Statistical analysis

The data were analyzed using SAS statistical software, version 9.0, (SAS Institute, Inc., Cary, NC, USA) and SmartPLS, version 2.0 M3 (SmartPLS, Hamburg, Germany). The proportion of MDR-TB in new cases was first calculated for each of the 120 counties. Spearman rank correlations were then computed to examine the bivariate correlations among risk factors in view of the non-normal characteristics of the data. Structural Equation Modeling (SEM) was also employed to explore the relationship between ecological risk factors and the proportion of MDR-TB. The obtained model was tested and modified using the PLS-PM approach. A *P*-value less than 0.10 was considered statistically significant.

**Fig 2:**
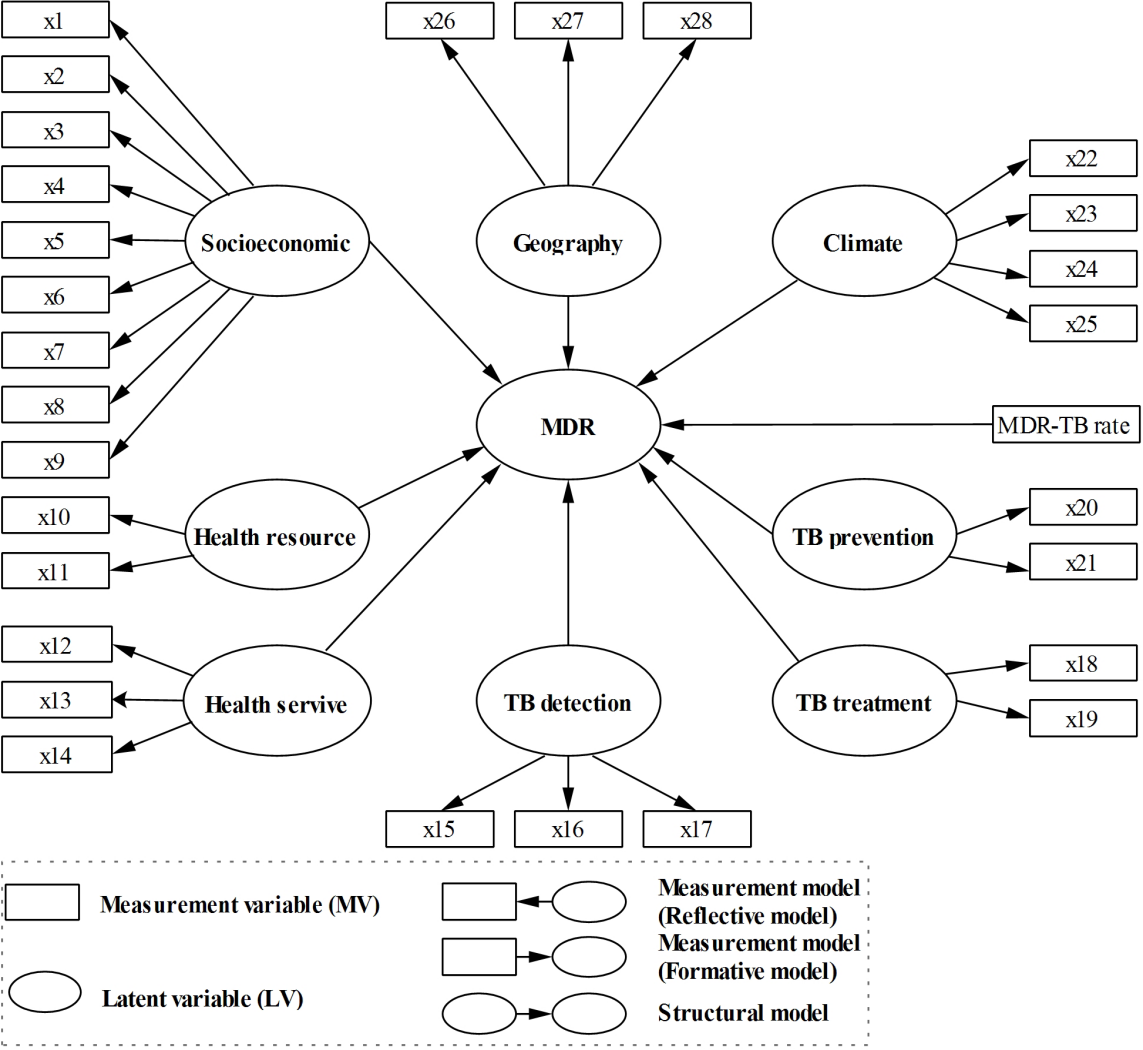
Initial PLS-PM of the prevalence of MDR-TB and related risk factors.

SEM can be viewed as a combination of factor analysis and multiple regression [31] including two parts: a measurement model relating the measurement variables (MVs) to their own latent variable (LV, the unmeasurable variable) and a structural model relating some LVs to other LVs. In this study, an exploratory factor analysis (EFA) [32,33] was first used to explore the latent structure of the risk-factor variables; then, an initial SEM (Fig 2) was constructed based on the results of the EFA. However, considering that the data were obviously characterized by not only skewed distribution but also small sample size and highly correlated variables, a PLS algorithm was used to test and modify the proposed model [34,35]. PLS-PM is an iterative algorithm that offers explicit estimates of the LVs using fewer cases and fewer assumptions about the data distribution. Measurement models can be either reflective or formative, depending on the causality between the MVs and LVs (Fig 2). A reflective model is used to construct the measurement model to extract the commonly encountered correlation structure between MVs, while a formative model is an alternative when only one MV exists [35]. In reflective blocks of PLS-PM, MV loadings indicate how well the indicators reflect its LV. In formative blocks, weights/loadings allow us to determine the extent to which each indicator contributed to the formation of the constructs.

For PLS-PM, no global goodness-of-fit criterion exists because it is assumed that the variance is distribution free. Alternatively, there is a set of standard measures for PLS-PM. The reliability and validity of the model estimation were evaluated according to these measures. For a reflective measurement model, composite reliability (CR), which measures internal consistency, is considered acceptable if its value is greater than 0.7 for established constructs [34,35]. Similarly, factor loadings, reflecting the MVs’ variance explained by the construct, are also considered acceptable when they are greater than 0.7 and should be eliminated when they are below 0.4 [36]. The average variance extracted (AVE) is used to evaluate the discriminant validity and should also be greater than 0.5 for all latent variables [34,35]. For a structural model, the path coefficients are evaluated first in terms of sign and significance by applying a bootstrapping test. The determination coefficient *R* ^2^ is then used to reflect the level or share of the composites’ explained variance. The SmartPLS software, which support graphical modeling, was used for the statistical analysis. A bootstrapping procedure was adopted to assess statistical significance. In this study, the inner estimate of the standardized latent variable was accomplished through a path-weighting scheme, and 1000 resamples were specified in the bootstrapping procedure.

## Results

### Prevalence of MDR-TB

Henan, Heilongjiang, Chongqing, Zhejiang and Tianjin started anti-TB drug resistance project in 2001, 2004, 2005, 2003 and 2008, respectively. The proportion of new TB cases across the 120 counties with MDR ranged from 0% to 39.39%. It presented obviously skewed (S1 Fig) with a median value of 3.96% and an interquartile range (IQR) of 1.52%-8.61%. Table 1 showed detailed data on MDR-TB in five provinces (municipalities).

**Table 1:** MDR-TB surveillance in 5 provinces (municipalities), China.

Province/municipality	Surveillance year	Surveillance number	Number of MDR-TB^a^ cases	Median proportion of MDR-TB (IQR^b^)
Henan	2002	1221	95	6.29%(2.30–12.20)
Heilongjiang	2004	1582	119	7.14%(3.99–10.00)
Chongqing	2005	957	26	3.03%(0.00–3.03)
Zhejiang	2003	777	35	3.57%(2.03–8.67)
Tianjin	2008	212	5	2.08%(0.00–4.50)

^a^Multidrug-resistant tuberculosis.

^b^Interquartile range.

### Ecological risk factors for MDR-TB

Twenty-eight risk factors were considered (Table 2), including socioeconomic, health resource allocation, health service, TB prevention and treatment, climate and geography factors. Table 3 presented the measurements of possible ecological risk factors associated with MDR-TB, with the median and IQR. A preliminary analysis showed that there were significant correlations between various risk-factors based on Spearman rank correlation (S1 Table). For instance, significant correlations were found among x2-x8 (socioeconomic variables) and x22-x25 (climate variables), suggesting that traditional regression models (e.g., ordinary linear regression) might be inappropriate.

**Table 2:** Latent factors and their corresponding risk factors.

Latent factors	Risk factors	
**Socioeconomic**	x1	Population density
	x2	Gross domestic product per capita
	x3	Per capita disposable income of urban residents
	x4	Per capita net income of rural residents
	x5	Per capita consumption of urban residents
	x6	Per capita consumption of rural residents
	x7	Per capita housing area of urban residents
	x8	Per capita housing area of rural residents
	x9	Life expectancy
**Health resource**	x10	Health institute beds per 1000 people
	x11	Medical technical personnel per 1000 people
**Health service**	x12	Child immunization rate (%)
	x13	Immunization coverage rate (%)
	x14	Infant mortality (‰)
**TB detection**	x15	Proportion of suspected TB patients treated
	x16	Notification rate of new smear-positive TB patients
	x17	Notification rate of re-treatment smear-positive TB patients
**TB treatment**	x18	Cure rate of new smear-positive TB patients
	x19	Cure rate of re-treatment smear-positive TB patients
**TB prevention**	x20	Personnel allocation for TB prevention and control per 100,000 people
	x21	Time interval between the directly observed therapy short course implementation and drug-resistant monitoring date
**Climate**	x22	Annual average temperature
	x23	Annual average humidity
	x24	Annual sunshine hours
	x25	Annual precipitation
**Geography**	x26	Annual average pressure
	x27	Proportion of mountains and hills (%)
	x28	Proportion of plains (%)

**Table 3:** The median (interquartile range) of ecological risk factors for MDR-TB in 5 provinces (municipalities).

Risk factors	Henan		Heilongjiang		Tianjin		Zhejiang		Chongqing	
**x1**	670.54	(476.21–801.63)	147.20	(81.33–297.32)	19105.50	(760.50–21498.75)	511.26	(326.45–759.75)	483.03	(335.09–672.96)
**x2**	5280.76	(3506.27–7156.02)	8018.00	(5983.50–10578.00)	34411.39	(21072.92–79107.79)	21760.00	(11707.00–26097.25)	9933.50	(6584.00–12461.75)
**x3**	4517.50	(3950.14–5131.5)	5808.45	(4499.25–6664.25)	19029.60	(17250.42–22941.25)	12601.00	(11965.75–13995.50)	9300.00	(8177.00–10255.50)
**x4**	2188.92	(2010.05–2513.02)	3227.50	(2903.75–3857.50)	8572.00	(8572.00–10431.50)	5802.00	(4553.25–6603.50)	3263.69	(2544.18–3637.53)
**x5**	3611.48	(3391.50–4273.22)	4782.00	(3954.75–6406.18)	12029.00	(12029.00–13422.00)	9645.00	(8798.91–10449.50)	8003.50	(6585.00–8623.29)
**x6**	1555.00	(1410.00–1999.40)	2340.65	(2060.25–3387.00)	4593.00	(4593.00–5746.25)	4775.00	(3622.25–5584.77)	2422.98	(2024.69–2713.80)
**x7**	20.96	(19.61–22.40)	15.94	(15.00–20.50)	27.09	(27.09–27.09)	25.05	(21.17–28.46)	23.00	(23.00–23.50)
**x8**	25.10	(25.10–25.15)	20.36	(20.36–20.40)	26.60	(26.30–26.60)	47.56	(44.49–54.87)	34.46	(32.45–38.76)
**x9**	71.93	(71.48–72.85)	73.00	(70.22–75.00)	79.41	(79.28–81.95)	75.24	(75.24–75.36)	73.76	(73.00–74.19)
**x10**	1.28	(0.99–1.56)	2.03	(1.21–4.64)	1.82	(1.40–3.99)	1.89	(1.61–2.53)	1.69	(1.21–2.31)
**x11**	2.08	(1.84–2.64)	2.58	(1.79–5.10)	2.44	(2.29–3.84)	1.32	(1.18–1.64)	2.22	(1.60–2.80)
**x12**	98.96	(98.00–99.75)	100.00	(98.69–100.00)	100.00	(99.63–100.00)	100.00	(99.81–100.00)	99.02	(96.55–100.00)
**x13**	96.90	(93.15–98.74)	99.45	(97.75–99.83)	99.08	(94.55–99.84)	98.10	(97.94–99.13)	94.88	(91.55–97.18)
**x14**	1.60	(1.17–2.24)	0.70	(0.48–0.93)	0.44	(0.38–0.68)	0.67	(0.61–0.84)	0.88	(0.64–1.27)
**x15**	0.19	(0.13–0.25)	0.24	(0.15–0.40)	0.08	(0.04–0.22)	0.07	(0.06–0.10)	0.29	(0.22–0.39)
**x16**	0.02	(0.01–0.02)	0.033	(0.03–0.05)	0.006	(0.002–0.015)	0.02	(0.017–0.027)	0.04	(0.03–0.05)
**x17**	0.004	(0.002–0.006)	0.011	(0.01–0.02)	0.0009	(0.0004–0.0018)	0.004	(0.002–0.005)	0.007	(0.004–0.01)
**x18**	0.92	(0.89–0.96)	0.91	(0.87–0.95)	0.83	(0.73–0.90)	0.90	(0.78–0.96)	0.92	(0.86–0.95)
**x19**	0.77	(0.54–0.90)	0.83	(0.76–0.90)	0.62	(0.37–0.78)	0.80	(0.69–0.88)	0.82	(0.69–0.92)
**x20**	1.46	(1.01–2.07)	2.28	(1.77–4.36)	2.47	(1.95–6.67)	0.80	(	1.27	(1.00–1.74)
**x21**	-5.53	(-6.29–5.03)	123.27	(114.41–131.93)	189.74	(118.44–267.59)	13.20	(7.15–74.05)	139.55	(95.92–144.88)
**x22**	15.15	(14.75–15.70)	4.65	(4.05–5.30)	13.30	(13.30–13.53)	18.15	(17.65–18.60)	18.40	(18.05–18.60)
**x23**	65.00	(63.00–73.63)	64.00	(60.00–67.5)	56.00	(56.00–59.00)	75.00	(72.41–78.00)	77.84	(74.90–79.08)
**x24**	2005.75	(1907.00–2154.30)	2545.10	(2449.10–2717.50)	2415.40	(2415.40–2415.40)	1931.90	(1833.78–1963.80)	1132.50	(980.05–1215.13)
**x25**	687.35	(570.39–880.35)	477.10	(389.13–527.83)	552.10	(552.10–612.18)	1363.45	(1001.63–1573.17)	1123.05	(1018.80–1230.77)
**x26**	1005.22	(996.05–1009.75)	992.70	(984.98–996.03)	1016.80	(1016.73–1016.80)	1012.95	(1007.15–1016.00)	981.95	(972.00–986.38)
**x27**	34.25	(0.00–85.26)	49.18	(0.00–77.55)	0.00	(0.00–0.00)	66.83	(42.56–76.78)	94.91	(92.03–97.09)
**x28**	65.76	(12.44–91.15)	41.76	(18.21–89.31)	74.00	(74.00–90.64)	21.55	(11.91–41.09)	0.00	(0.00–2.11)

### The latent structure of the risk factor variables

Six latent factors (‘Socioeconomic’, ‘Health resource’, ‘Health service’, ‘TB detection’, ‘TB treatment’ and ‘TB prevention’) were extracted by an EFA from risk factors *x*1-*x*21 (Table 2). Together, these factors explained approximately 72.56% of the total variance for the 21 variables. Two latent factors (‘Climate’ and ‘Geography’) were extracted from risk factors *x*22-*x*28 (Table 2), and they explained 74.23% of the total variance. Table 2 describes the latent factors and the corresponding risk factors in detail. From the table, we can clearly make the following observations: the higher the score on the ‘Socioeconomic’ factor, the higher the socioeconomic level; the higher the score on the ‘Health resource’ factor, the better the allocation of health resources; the higher the score on the ‘Health service’ factor, the lower the health service level; the higher the score on the ‘TB detection’ factor, the better the TB detection work; the higher the score on the ‘TB treatment’ factor, the higher the TB cure rate; the higher the score on the ‘TB prevention’ factor, the better the TB prevention; the higher the score on the ‘Climate’ factor, the more humid the climate; and the higher the score on the ‘Geography’ factor, the higher the elevation.

### Complex relationship between the proportion of MDR-TB and risk factors

**Fig 3:**
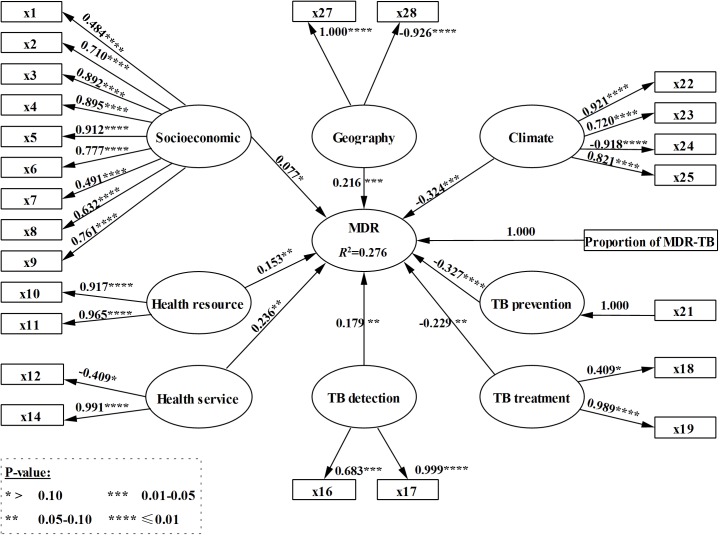
Modified PLS-PM of the prevalence of MDR-TB and related risk factors.

Based on the results of the EFA, we constructed an initial model of the proportion of MDR-TB with risk factors (Fig 2). Based on the iterative PLS-PM procedure and the practical meanings of variables, four MVs (x13, x15, x20, and x26) were removed. The modified model depicted in Fig 3 included the bootstrapping test results (*P*-values) for the loadings of the measurement model and path coefficients of the structure model. Table 4 presents the evaluation of the measurement model, which showed that all factor loadings were higher than 0.4, the CR for each LV was above or close to 0.7, and the AVE was always greater than 0.5. Therefore, the measurement models were considered acceptable for evaluation of the structural model. The *R* ^2^ for the model was 0.276, indicating that 27.6% of the total variance in the proportion of MDR-TB was explained by the risk-factor variables.

**Table 4:** Evaluation of the measurement model.

LVs^a^	MVs^b^	Loading	CR^c^	AVE^d^
**MDR**	Proportion of MDR_TB	1.000	1.000	1.000
**Socioeconomic**	x1	0.484	0.915	0.554
	x2	0.710		
	x3	0.892		
	x4	0.895		
	x5	0.912		
	x6	0.777		
	x7	0.491		
	x8	0.632		
	x9	0.761		
**Health resource**	x10	0.917	0.939	0.886
	x11	0.965		
**Health service**	x12	-0.409	0.698	0.575
	x14	0.991		
**TB detection**	x16	0.683	0.841	0.732
	x17	0.999		
**TB treatment**	x18	0.409	0.696	0.572
	x19	0.989		
**TB prevention**	x21	1.000	1.000	1.000
**Climate**	x22	0.921	0.681	0.721
	x23	0.720		
	x24	-0.918		
	x25	0.821		
**Geography**	x27	1.000	0.963	0.929
	x28	-0.926		

^a^latent variables.

^b^measurement variables.

^c^composite reliability.

^d^average variance extracted.

In the modified PLS-PM (Fig 3), the remaining variables had a substantial relationship with their respective dependent variables. All of the path coefficients, interpreted as standardized beta coefficients, were statistically significant (*P*≤0.10) except for the path from ‘Socioeconomic’ to ‘MDR.’ Nevertheless, this path met the evaluation criteria and was retained. The ‘TB prevention’ factor had the largest effect, with a standardized path coefficient of -0.327 (i.e., there was a negative relationship between the ‘TB prevention’ and ‘MDR’ factors). Additionally, the ‘TB treatment’ and ‘Climate’ factors both had negative relationships with ‘MDR’ with standard path coefficients of -0.229 and -0.324, respectively. Finally, the ‘Health Resources’, ‘Health Services’, ‘TB Detection’ and ‘Geography’ factors all had positive relationships with the ‘MDR’ factor, with standardized path coefficients of 0.153, 0.236, 0.179 and 0.216, respectively.

## Discussion

Our findings suggest that “TB prevention”, “health resources”, “health services”, “TB treatment”, “TB detection”, “geography” and “climate” factors affect “MDR” significantly. In this study, 120 counties in Tianjin, Chongqing, Zhejiang, Heilongjiang and Henan, with 4,749 new TB cases, were included. Each of these five provinces (or municipalities) designed and conducted drug resistance surveillance programs based on the methods and standards in the WHO/IUATLD Guidelines for surveillance of drug resistance [6] and adopted strict quality control measures. Although many studies have explored individual-level risk factors for MDR-TB, such as age, sex genetic susceptibility, occupation, previous treatment, smoking, HIV infection and socioeconomic factors [7–25], few studies have addressed group-level risk factors. Therefore, we focused on group-level risk factors associated with MDR-TB.

Eight latent factors (‘Socioeconomic’, ‘Health resource’, ‘Health service’, ‘TB detection’, ‘TB treatment’, ‘TB prevention’, ‘Climate’ and ‘Geography’) were extracted and included in the modified PLS-PM, which explained 27.6% of the total variance in the prevalence of MDR-TB. All of these risk factors exerted a significant influence on the development of MDR-TB with the exception of ‘Socioeconomic’. Based on our data, socioeconomic factors were not found to be associated with the development of MDR-TB. However, some studies of individual-level risk factors for MDR-TB had reported that socioeconomic factors were related to MDR-TB [15,25,24,]. This apparent discrepancy may be a result of the different levels of analysis (individual-level or group-level) and different populations. In some ecological studies for TB risk factors, socioeconomic factors were also found to be correlated with the development of TB [37,38]. ‘TB prevention’ and ‘TB treatment’ both exhibited a negative relationship with ‘MDR’ in this study; i.e., the earlier the directly observed therapy short course implementation and the higher the TB cure rate, the lower the prevalence of MDR-TB. This result is similar to the finding from a national survey of drug-resistant TB in China that underscored the need for intervention that will increase continuity of treatment and reduce the rate of treatment default [39]. In contrast, ‘Health resource’, ‘Health service’ and ‘TB detection’ each had a positive relationship with the ‘MDR’ factor, indicating that the more suitable the allocation of health resources and the higher the health service level, the higher the prevalence of MDR-TB. This finding can be attributed, at least in part, to the elevated discovery rate of MDR-TB, presumably as a function of better health resources and health services. Note that the ‘Health service’ score runs contrary to the others. The reason for this finding is that the latent factor ‘Health service’ had a negative correlation with the child immunization rate and had a positive correlation with infant mortality, so higher scores on the ‘Health service’ factor implied higher infant mortality and a lower child immunization rate, i.e., a lower level of health services. ‘Climate’ had a negative relationship with ‘MDR’, i.e., the more humid the climate, the lower the prevalence of MDR-TB. The ‘Geography’ factor had a positive relationship with ‘MDR’, i.e., the higher the elevation, the higher the prevalence of MDR-TB. A previous study based on “Anti-tuberculosis drug resistance in the world: Report no. 4” also showed that some ecological factors associated with DR-TB, such as health expenditure, humidity and temperature (with a negative relationship) and TB epidemic, health expenditure and the directly observed therapy short course (with a positive effect) [27]. Another study of MDR-TB trends based on surveillance and population-representative survey data collected worldwide by the WHO found that better surveillance indicators and a higher GDP per capita were associated with declining MDR-TB, whereas a higher existing absolute burden of MDR-TB was associated with an increasing trend [40]. Therefore, a reasonable MDR-TB monitoring plan, as well as prevention and control strategies, should be formulated based on the relationship between the prevalence of MDR-TB and the related risk factors, e.g., increasing health resources, improving health service levels and DOTS implementation.

PLS-PM was used to construct an SEM for the relationship between ecological risk factors and MDR-TB. The variables typically exhibited not only multicollinearity but also non-normality and a small sample size. As is well known, traditional multivariate regression may perform poorly on datasets with multiple independent variables showing multicollinearity. Moreover, although some machine learning methods such as the support vector machine method are not heavily affected by multicollinearity, it appears to be unable to synthesize the manifest variables to obtain a latent score. PLS-PM is a “soft modeling” approach, requiring fewer distributional assumptions, and the variables studied can be numerical, ordinal, or nominal; hence, the procedure requires no normality assumptions [34,35]. This is a very appealing feature for the analysis of the ecological risk factors involved in this study. Thus, taking the anti-TB drug resistance monitoring county as the unit of analysis, we employed an EFA to identify the LVs and explore the relationship between ecological risk factors and MDR-TB prevalence levels using PLS-PM.

This study had several limitations. First, we only focused on MDR-TB in new cases rather than retreatment cases; the reason was that few retreatment cases were detected in each province. Thus, we did not cover acquired and transmitted resistance MDR-TB cases. Second, information on HIV infection status for TB cases was not collected here because TB patients in China are not routinely tested for HIV.

In conclusion, this study expands the current knowledge of the ecological risk factors for MDR-TB in China. The results showed that TB prevention, health resources, health services, TB treatment, TB detection, geography and climate factors were associated with the occurrence of multidrug resistance. Despite the limitations mentioned above, understanding the risk factors associated with MDR-TB can provide context for future studies that will yield benefits to the entire public health system in China.

## Supporting Information

S1 Fig The distribution of the proportion of MDR-TB in new TB cases in 120 counties. (TIF)

S1 Table Correlations among risk-factor variables. (DOCX)

S1 Dataset Data on MDR-TB and ecological risk factors at the county level. (XLS)
