# Supplementary figures and images for: Association between Multidrug-Resistant Tuberculosis and Risk Factors in China: Applying Partial Least Squares Path Modeling
Source: PLoS One. 2015 May 28;10(5):e0128298. doi: 10.1371/journal.pone.0128298 (PMC4447294; doi:10.1371/journal.pone.0128298)

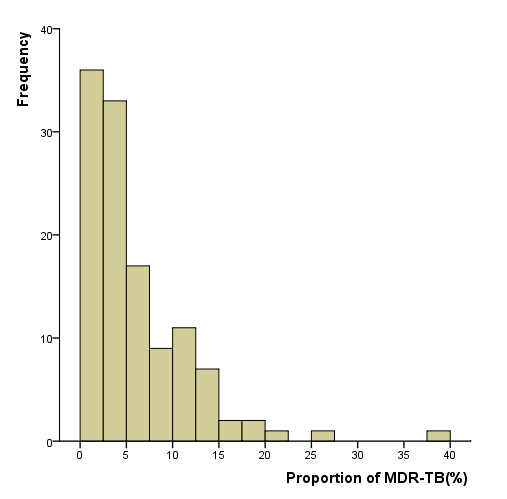

Supplement: S1 Fig — (TIF) [file pone.0128298.s001.tif]
