# Supplementary material for: Association between Multidrug-Resistant Tuberculosis and Risk Factors in China: Applying Partial Least Squares Path Modeling
Source: PLoS One. 2015 May 28;10(5):e0128298. doi: 10.1371/journal.pone.0128298 (PMC4447294; doi:10.1371/journal.pone.0128298)
Supplement: S1 Table — (DOCX) [file pone.0128298.s002.docx]

**S1 Table. Correlations among risk-factor variables.**

|  | x1 | x2 | x3 | x4 | x5 | x6 | x7 | x8 | x9 | x10 | x11 | x12 | x13 | x14 | x15 | x16 | x17 | x18 | x19 | x20 | x21 | x22 | x23 | x24 | x25 | x26 | x27 | x28 |
| --- | --- | --- | --- | --- | --- | --- | --- | --- | --- | --- | --- | --- | --- | --- | --- | --- | --- | --- | --- | --- | --- | --- | --- | --- | --- | --- | --- | --- |
| x1 | 1 |  |  |  |  |  |  |  |  |  |  |  |  |  |  |  |  |  |  |  |  |  |  |  |  |  |  |  |
| x2 | 0.322^‡^ | 1 |  |  |  |  |  |  |  |  |  |  |  |  |  |  |  |  |  |  |  |  |  |  |  |  |  |  |
| x3 | 0.297^‡^ | 0.745^‡^ | 1 |  |  |  |  |  |  |  |  |  |  |  |  |  |  |  |  |  |  |  |  |  |  |  |  |  |
| x4 | 0.332^‡^ | 0.891^‡^ | 0.778^‡^ | 1 |  |  |  |  |  |  |  |  |  |  |  |  |  |  |  |  |  |  |  |  |  |  |  |  |
| x5 | 0.316^‡^ | 0.747^‡^ | 0.870^‡^ | 0.776^‡^ | 1 |  |  |  |  |  |  |  |  |  |  |  |  |  |  |  |  |  |  |  |  |  |  |  |
| x6 | 0.170^*^ | 0.771^‡^ | 0.648^‡^ | 0.821^‡^ | 0.711^‡^ | 1 |  |  |  |  |  |  |  |  |  |  |  |  |  |  |  |  |  |  |  |  |  |  |
| x7 | 0.288^‡^ | 0.244^‡^ | 0.451^‡^ | 0.231^†^ | 0.432^‡^ | 0.208^†^ | 1 |  |  |  |  |  |  |  |  |  |  |  |  |  |  |  |  |  |  |  |  |  |
| x8 | 0.309^‡^ | 0.508^‡^ | 0.640^‡^ | 0.430^‡^ | 0.639^‡^ | 0.422^‡^ | 0.583^‡^ | 1 |  |  |  |  |  |  |  |  |  |  |  |  |  |  |  |  |  |  |  |  |
| x9 | 0.187^†^ | 0.566^‡^ | 0.630^‡^ | 0.585^‡^ | 0.590^‡^ | 0.524^‡^ | 0.295^‡^ | 0.418^‡^ | 1 |  |  |  |  |  |  |  |  |  |  |  |  |  |  |  |  |  |  |  |
| x10 | 0.189^†^ | 0.574^‡^ | 0.386^‡^ | 0.555^‡^ | 0.335^‡^ | 0.406^‡^ | 0.038 | 0.136 | 0.256^‡^ | 1 |  |  |  |  |  |  |  |  |  |  |  |  |  |  |  |  |  |  |
| x11 | 0.161^*^ | 0.063 | -0.183^†^ | 0.034 | -0.190^†^ | -0.053 | -0.233^‡^ | -0.392^‡^ | -0.129 | 0.506^‡^ | 1 |  |  |  |  |  |  |  |  |  |  |  |  |  |  |  |  |  |
| x12 | 0.022 | 0.312^‡^ | 0.264^‡^ | 0.424^‡^ | 0.278^‡^ | 0.355^‡^ | -0.07 | 0.134 | 0.337^‡^ | 0.204^†^ | 0.043 | 1 |  |  |  |  |  |  |  |  |  |  |  |  |  |  |  |  |
| x13 | 0.022 | 0.303^‡^ | 0.096 | 0.327^‡^ | 0.109 | 0.256^‡^ | -0.255^‡^ | -0.156^*^ | 0.211^†^ | 0.236^‡^ | 0.133 | 0.353^‡^ | 1 |  |  |  |  |  |  |  |  |  |  |  |  |  |  |  |
| x14 | -0.071 | -0.498^‡^ | -0.418^‡^ | -0.571^‡^ | -0.401^‡^ | -0.454^‡^ | -0.016 | -0.065 | -0.344^‡^ | -0.380^‡^ | -0.066 | -0.309^‡^ | -0.281^‡^ | 1 |  |  |  |  |  |  |  |  |  |  |  |  |  |  |
| x15 | -0.264^‡^ | -0.313^‡^ | -0.305^‡^ | -0.393^‡^ | -0.365^‡^ | -0.350^‡^ | -0.156^*^ | -0.411^‡^ | -0.314^‡^ | -0.081 | 0.208^†^ | -0.196^†^ | -0.092 | 0.087 | 1 |  |  |  |  |  |  |  |  |  |  |  |  |  |
| x16 | -0.410^‡^ | -0.086 | 0.02 | -0.077 | -0.016 | -0.002 | -0.107 | -0.175^†^ | -0.127 | 0.107 | 0.028 | -0.040 | -0.027 | -0.11 | 0.577^‡^ | 1 |  |  |  |  |  |  |  |  |  |  |  |  |
| x17 | -0.429^‡^ | -0.286^‡^ | -0.231^†^ | -0.273^‡^ | -0.304^‡^ | -0.184^†^ | -0.287^‡^ | -0.447^‡^ | -0.213^†^ | -0.011 | 0.127 | -0.068 | 0.061 | -0.015 | 0.577^‡^ | 0.633^‡^ | 1 |  |  |  |  |  |  |  |  |  |  |  |
| x18 | -0.106 | -0.067 | -0.169^*^ | -0.102 | -0.193^†^ | -0.136 | -0.086 | -0.057 | -0.084 | 0.078 | 0.058 | -0.139 | -0.170^*^ | -0.010 | 0.074 | 0.124 | 0.036 | 1 |  |  |  |  |  |  |  |  |  |  |
| x19 | -0.154^*^ | -0.105 | -0.131 | -0.195^†^ | -0.161^*^ | -0.142 | -0.12 | -0.111 | -0.118 | 0.024 | -0.014 | -0.132 | -0.135 | 0.030 | 0.145 | 0.259^‡^ | 0.259^‡^ | 0.588^‡^ | 1 |  |  |  |  |  |  |  |  |  |
| x20 | -0.247^‡^ | -0.217^†^ | -0.212^†^ | -0.190^†^ | -0.204^†^ | -0.254^‡^ | -0.182^†^ | -0.580^‡^ | -0.125 | 0.046 | 0.327^‡^ | -0.084 | 0.116 | -0.158^*^ | 0.368^‡^ | 0.246^‡^ | 0.311^‡^ | -0.003 | 0.062 | 1 |  |  |  |  |  |  |  |  |
| x21 | -0.269^‡^ | 0.232^†^ | 0.270^‡^ | 0.248^‡^ | 0.260^‡^ | 0.267^‡^ | 0.067 | -0.140 | 0.208^†^ | 0.190^†^ | 0.156^*^ | 0.096 | 0.070 | -0.326^‡^ | 0.270^‡^ | 0.491^‡^ | 0.284^‡^ | -0.110 | 0.020 | 0.281^‡^ | 1 |  |  |  |  |  |  |  |
| x22 | 0.272^‡^ | 0.272^‡^ | 0.442^‡^ | 0.158^*^ | 0.452^‡^ | 0.201^†^ | 0.544^‡^ | 0.842^‡^ | 0.244^‡^ | 0.010 | -0.368^‡^ | -0.078 | -0.297^‡^ | 0.111 | -0.151^*^ | 0.002 | -0.319^‡^ | -0.028 | 0.013 | -0.563^‡^ | -0.105 | 1 |  |  |  |  |  |  |
| x23 | -0.015 | 0.118 | 0.314^‡^ | 0.044 | 0.240^‡^ | 0.076 | 0.356^‡^ | 0.567^‡^ | 0.040 | 0.008 | -0.301^‡^ | -0.033 | -0.223^†^ | 0.096 | 0.031 | 0.197^†^ | 0.033 | -0.026 | 0.028 | -0.367^‡^ | 0.009 | 0.627^‡^ | 1 |  |  |  |  |  |
| x24 | -0.246^‡^ | -0.079 | -0.257^‡^ | 0.053 | -0.226^†^ | 0.037 | -0.473^‡^ | -0.699^‡^ | -0.075 | -0.011 | 0.183^†^ | 0.127 | 0.478^‡^ | -0.194^†^ | -0.007 | -0.070 | 0.177^†^ | -0.026 | -0.027 | 0.497^‡^ | 0.076 | -0.812^‡^ | -0.656^‡^ | 1 |  |  |  |  |
| x25 | 0.169^*^ | 0.208^†^ | 0.461^‡^ | 0.155^*^ | 0.455^‡^ | 0.238^‡^ | 0.500^‡^ | 0.795^‡^ | 0.237^‡^ | -0.028 | -0.432^‡^ | 0.021 | -0.302^‡^ | 0.127 | -0.205^†^ | 0.008 | -0.249^‡^ | -0.026 | 0.015 | -0.524^‡^ | -0.149 | 0.814^‡^ | 0.730^‡^ | -0.728^‡^ | 1 |  |  |  |
| x26 | 0.395^‡^ | 0.235^‡^ | 0.335^‡^ | 0.336^‡^ | 0.333^‡^ | 0.256^‡^ | 0.182^†^ | 0.257^‡^ | 0.302^‡^ | -0.047 | -0.313^‡^ | 0.121 | 0.066 | -0.091 | -0.526^‡^ | -0.482^‡^ | -0.374^‡^ | -0.09 | -0.143 | -0.168^*^ | -0.305^‡^ | 0.042 | -0.109 | 0.159^*^ | 0.106 | 1 |  |  |
| x27 | -0.264^‡^ | -0.057 | 0.078 | -0.156^*^ | 0.078 | -0.026 | 0.210^†^ | 0.288^‡^ | -0.148 | -0.012 | -0.040 | -0.129 | -0.294^‡^ | 0.148 | 0.234^‡^ | 0.393^‡^ | 0.146 | 0.102 | 0.097 | -0.196^†^ | 0.151 | 0.461^‡^ | 0.433^‡^ | -0.539^‡^ | 0.452^‡^ | -0.527^‡^ | 1 |  |
| x28 | 0.132 | -0.115 | -0.219^†^ | -0.022 | -0.201^†^ | -0.1 | -0.249^‡^ | -0.387^‡^ | -0.008 | -0.117 | -0.029 | 0.021 | 0.244^‡^ | -0.049 | -0.214^†^ | -0.368^‡^ | -0.111 | -0.088 | -0.055 | 0.230^†^ | -0.221^†^ | -0.534^‡^ | -0.464^‡^ | 0.598^‡^ | -0.463^‡^ | 0.484^‡^ | -0.901^‡^ | 1 |
